# Gallbladder polyps: Correlation of size and clinicopathologic characteristics based on updated definitions

**DOI:** 10.1371/journal.pone.0237979

**Published:** 2020-09-11

**Authors:** Orhun C. Taskin, Olca Basturk, Michelle D. Reid, Nevra Dursun, Pelin Bagci, Burcu Saka, Serdar Balci, Bahar Memis, Enrique Bellolio, Juan Carlos Araya, Juan Carlos Roa, Oscar Tapia, Hector Losada, Juan Sarmiento, Kee-Taek Jang, Jin-Young Jang, Burcin Pehlivanoglu, Mert Erkan, Volkan Adsay

**Affiliations:** 1 Department of Pathology and Research Center for Translational Medicine (KUTTAM), Koç University, Istanbul, Turkey; 2 Memorial Sloan Kettering Cancer Center, Department of Pathology, New York, NY, United States of America; 3 Department of Pathology, Emory University, Atlanta, GA, United States of America; 4 Department of Pathology, Istanbul Research and Training Hospital, Istanbul, Turkey; 5 Department of Pathology, Marmara University Pendik Research and Training Hospital, Istanbul, Turkey; 6 Department of Pathology, Medipol University, Istanbul, Turkey; 7 Department of Pathology, Şişli Hamidiye Etfal Training and Research Hospital, Istanbul, Turkey; 8 Anatomic Pathology Department, Universidad de La Frontera, Temuco, Chile; 9 Department of Pathology, Hospital Dr. Hernan Henriquez Aravena, Temuco, Chile; 10 Department of Pathology, Pontificia Universidad Catolica de Chile, Santiago, Chile; 11 Department of Surgery and Traumatology, Universidad de La Frontera, Temuco, Chile; 12 Department of Surgery, Emory University, Atlanta, GA, United States of America; 13 Department of Surgery, Seoul National University College of Medicine, Seoul, Korea; 14 Department of Pathology, Adiyaman Training and Research Hospital, Adiyaman, Turkey; 15 Department of Surgery and Research Center for Translational Medicine (KUTTAM), Koç University Hospital, Istanbul, Turkey; Universita degli Studi di Verona, ITALY

## Abstract

**Background:**

Different perspectives exist regarding the clinicopathologic characteristics, biology and management of gallbladder polyps. Size is often used as the surrogate evidence of polyp behavior and size of ≥1cm is widely used as cholecystectomy indication. Most studies on this issue are based on the pathologic correlation of polyps clinically selected for resection, whereas, the data regarding the nature of polypoid lesions from pathology perspective -regardless of the cholecystectomy indication- is highly limited.

**Methods:**

In this study, 4231 gallbladders -606 of which had gallbladder carcinoma- were reviewed carefully pathologically by the authors for polyps (defined as ≥2 mm). Separately, the cases that were diagnosed as “gallbladder polyps” in the surgical pathology databases were retrieved.

**Results:**

643 polyps identified accordingly were re-evaluated histopathologically. Mean age of all patients was 55 years (range: 20–94); mean polyp size was 9 mm. Among these 643 polyps, 223 (34.6%) were neoplastic: I. Non-neoplastic polyps (n = 420; 65.4%) were smaller (mean: 4.1 mm), occurred in younger patients (mean: 52 years). This group consisted of fibromyoglandular polyps (n = 196) per the updated classification, cholesterol polyps (n = 166), polypoid pyloric gland metaplasia (n = 41) and inflammatory polyps (n = 17). II. Neoplastic polyps were larger (mean: 21 mm), detected in older patients (mean: 61 years) and consisted of intra-cholecystic neoplasms (WHO’s “adenomas” and “intracholecystic papillary neoplasms”, ≥1 cm; n = 120), their “incipient” version (<1 cm) (n = 44), polypoid invasive carcinomas (n = 26) and non-neoplastic polyps with incidental dysplastic changes (n = 33). In terms of size cut-off correlations, overall, only 27% of polyps were ≥1 cm, 90% of which were neoplastic. All (except for one) ≥2 cm were neoplastic. However, 14% of polyps <1 cm were also neoplastic. Positive predictive value of ≥1 cm cut-off -which is widely used for cholecystectomy indication-, was 94.3% and negative predictive value was 85%.

**Conclusions:**

Approximately a third of polypoid lesions in the cholecystectomies (regardless of the indication) prove to be neoplastic. The vast majority of (90%) of polyps ≥1 cm and virtually all of those ≥2 cm are neoplastic confirming the current impression that polyps ≥1 cm ought to be removed. However, this study also illustrates that 30% of the neoplastic polyps are <1 cm and therefore small polyps should also be closely watched, especially in older patients.

## Introduction

Polyps of the gallbladder are relatively common [1,2]. Most are detected during radiologic examination of the gallbladder, performed to investigate either symptoms attributable to the gallbladder itself, or other abdominal pathology. In some countries like Japan, ultrasonographic examination of the gallbladder, including the mucosal thickness, is part of the routine healthcare check-up mandated by the government, which leads to incidental discovery of gallbladder polyps as well.

Substantial changes have taken place in the terminology, classification and our understanding regarding the nature of polypoid lesions in the gallbladder in the past decade. The two broad categories established by Yamamoto et al since 1980s as neoplastic versus non-neoplastic (the latter with “hyperplastic” and “metaplastic” subsets) were expanded and modified over the years [3]. For the non-neoplastic group, in addition to well-recognized cholesterol polyps, mucosal injury polyps, including fibromyoglandular polyps were recognized [4]. A variety of other polyp types such as inflammatory fibroid polyp and others were discovered to occur in this organ [5,6]. For the neoplastic polyps, which were regarded in various different categories (i.e., pyloric gland adenoma, biliary adenoma, intestinal adenoma, tubular adenoma, tubulopapillary adenoma, papillary adenoma, papillary neoplasm, and papillary carcinoma) [7] are now collected under two headings in the WHO 2019 classification as “intracholecystic papillary neoplasms” (for the papillary examples) and “pyloric gland adenomas” (for the tubular ones with pyloric type glands) [8]. Because of the overlap between these two entities, the unifying term of “intracholecystic papillary tubular neoplasm” was proposed for these lesions, which are essentially gallbladder kindreds of intraductal neoplasms of the pancreas and biliary tract [9]. All of these pre-invasive neoplastic polyps can be described as “intracholecystic neoplasms”. Regardless of the terminology, which remains somewhat controversial, it is now widely agreed upon that these intracholecystic neoplasms (tumor-forming preinvasive adenomatous neoplasms) have a high incidence of association with (or progression) into carcinoma and thus, warrant early intervention.

The aforementioned developments have allowed better appreciation of the biologic behavior of distinct polyp types of this organ. However, question remains as to how these different entities manifest at the clinical level. In the daily practice, while making a decision of gallbladder removal, polyp size of ≥1 cm is the arbitrary rule-of-thumb criterion widely used to determine indication for cholecystectomy [2,10–14] although the validity of this is questioned by some [15]. This is partly because studies thus far have been mostly based on radiologically recognized and removed polyps, and relied mostly on pathology reports [16]. On the other hand, the correlation of polyp size with clinicopathologic parameters has not been systematically verified from the histopathology perspective on cholecystectomies indifferent to the clinical indication (without the selection bias) and by applying the recently modified pathologic criteria in classification of neoplastic and non-neoplastic lesions.

In this study, all the polypoid lesions that had been histopathologically recognized and classified by the authors using the current criteria were investigated to determine the clinicopathologic associations of polyps with different size.

## Methods

### Ethics statement

This retrospective study was performed in accordance with the institutional review board requirements and with the Helsinki Declaration and its later amendments or comparable standards. All data were fully anonymized before accession. [Emory University Hospitals, Emory University Institutional Review Board, granted on August 2008 (IRB00010713, date range: September 2008-April 2017); Hospital Dr. Hernán Henríquez Aravena de Temuco Chile, Comité de Evaluación Científica del Servicio de Salud Araucanía Sur, granted on April 2017 (DI17-0166, date range: 2006–2017)].

### Definition of polyp, inclusion criteria, selection of size cut-off

A polyp was defined as a protrusion of the mucosa that is clearly recognizable either on the gross bench or by examination of the glass slide and that formed a morphologically distinct lesion, with internal characteristics different than that of the neighboring structures as verified by microscopic examination. Whether this was recognized pre-operatively during radiologic examination was not taken into consideration, since the cases were identified through highly different criteria and had undergone different levels of pre-op radiologic work-up with variable sensitivities, and the purpose of this study was to determine the associations of polyps that were identified pathologically (not clinically). In other words, the polyps were defined histopathologically.

At the beginning of this study, our purpose was to analyze all histopathologically definable polyps regardless of the size. However, it became clear early on that most gallbladders, especially those with gallstones and injury, has some mucosal granularity and nodularity, which can technically qualify as a polyp. Therefore, a more specific definition -with a quantitative minimum size criterion- was needed. Along those lines, since a polyp is by definition an “elevated” lesion from the surface, some numeric criteria was required to define this status of “elevation”. Similarly, in order for a lesion to be recognizable as a pathologic abnormality, a zonal change that distinguishes it from the neighboring structures was also thought to be important, and this also warranted a quantitative measurement. Additionally, the purpose of the study was to determine the correlation of the size with clinicopathologic characteristics and therefore a minimum size criterion was needed for this reason as well. The 2 mm size cut-off was chosen based on the communication with radiologists who made clear that this is the smallest size recognizable safely and reproducibly by current imaging modalities.

### Exclusion criteria

Protrusions that measured less than 2 mm were excluded. Neoplastic or non-neoplastic lesions that had a flat appearance rather than polypoid were excluded by definition. Accordingly, since this study was purposefully blinded to the radiologic findings and focused on the pathology perspective of polyps instead, mural lesions that mimic polyps such as adenomyomas or inflammatory pseudotumors -that can be erroneously mistaken as “polyp” in radiologic studies [16]- were excluded. Instead, only lesions that had true mucosal polyp formation were analyzed with the assumption that with improving technology, radiologic techniques will allow the distinction of the true polyps from the mimickers.

### Histopathologic classification

The polyps were classified based on the recent updates on classification schemes [4,8,9,14].

I Non-neoplastic polyps:
  These were defined according to the criteria published recently [4,17,18].
  - Fibromyoglandular polyp: Broad-based polyps, mostly associated with gallstones and prominent inflammation, composed of lobules of small glandular structures, separated by fibroblastic and muscular stroma.
  - Cholesterol polyp: Pedunculated polyps with a unique cauliflower-like architecture, lined by single-layered normal gallbladder epithelium with widened edematous cores mostly devoid of glands.
  - Polypoid pyloric gland metaplasia: Polypoid mucosa with regenerative changes, harboring compact collection of metaplastic pyloric glands forming small protrusions.
  - Inflammatory polyp: Polyps composed entirely of prominent lymphoid aggregates and/or granulation tissue and/or xanthogranulomas.

**Fig 1:**
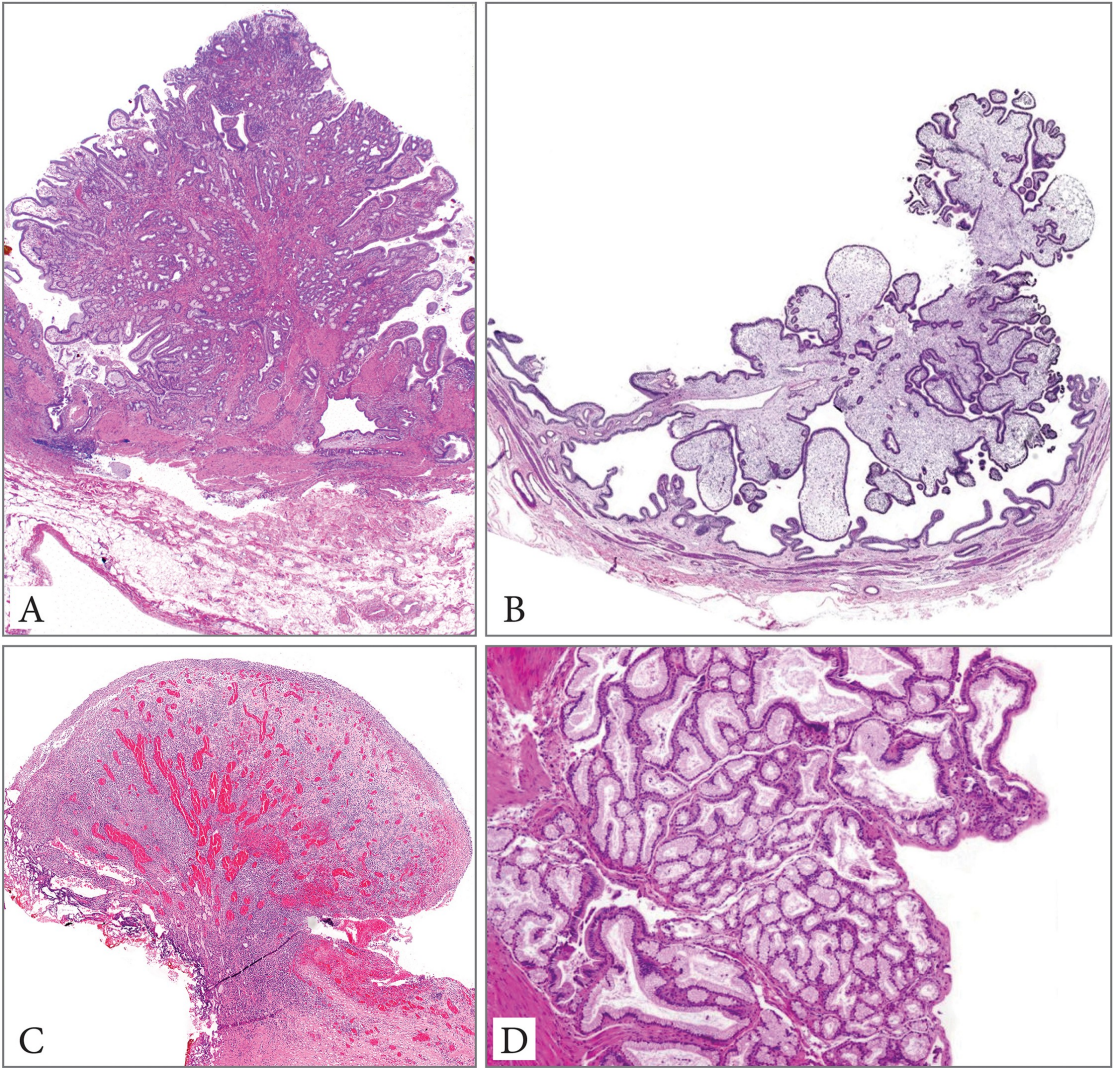
Non-neoplastic polyps: Fibromyoglandular polyp (A), cholesterol polyp (B), inflammatory polyp (C) and polypoid pyloric gland metaplasia (D) (Hematoxylin&eosin, 10x magnification).

See Fig 1 for different types of non-neoplastic polyps.

II Neoplastic polyps:
  - Intracholecystic neoplasms: For the purposes of this study, for the sake of simplicity, all mass-forming pre-invasive (tumoral intraepithelial) neoplasms including WHO’s “pyloric gland adenomas” and “intracholecystic papillary neoplasms” [8], as well as those cases reported under the heading of intracholecystic papillary tubular neoplasms [9] that formed a polyp ≥1 cm were regarded under the intracholecystic neoplasms category. These are, in essence, gallbladder counterparts of intraductal neoplasia of the pancreatobiliary tract, encompassing the lesion types morphologically similar to intraductal papillary neoplasm of the bile duct and intraductal papillary mucinous neoplasm of the pancreas, intraampullary papillary-tubular neoplasm [19], intraductal tubulopapillary neoplasm of the bile ducts [20] and pancreas [21], intraductal oncocytic papillary neoplasm of bile ducts and pancreas [22,23].
  - “Incipient” intracholecystic neoplasms: Lesions that histomorphologically qualify as intracholecystic neoplasms described above (that form compact polypoid lesion composed of dysplastic cells growing back-to-back and forming an adenomatous lesion) but measuring <1 cm.
  - Polypoid invasive carcinoma: Invasive carcinomas that grow in polypoid configuration in which polyp component is also invasive, not pre-invasive (i.e., not a dysplastic or an adenomatous lesion).
  - Non-neoplastic polyps harboring dysplasia per recently updated criteria [8,24,25].

**Fig 2:**
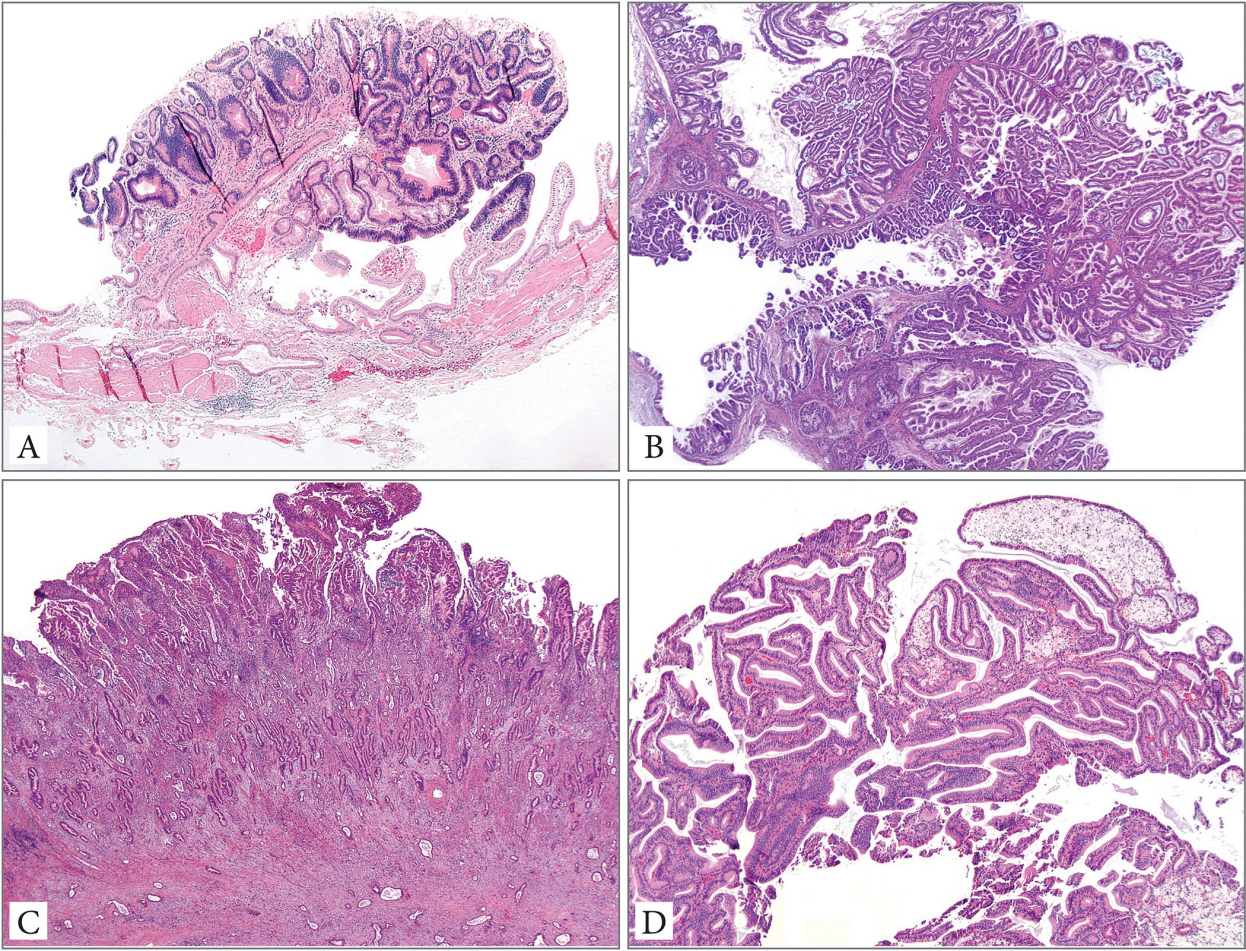
Neoplastic polyps: Incipient intracholecystic neoplasm (<1 cm by definition) (A), intracholecystic neoplasm (B), polypoid invasive adenocarcinoma (C) and non-neoplastic polyp (in this case, a cholesterol polyp) with low grade dysplasia (Hematoxylin&eosin, 10x magnification) (D).

See Fig 2 for different types of neoplastic polyps.

### Case population / databases

To retrieve polyps, a computer search of the pathology databases from 1994 to 2013 was conducted. Separately, all the slides and pathology material of 3715 consecutive routine cholecystectomies (with cholecystitis and/or gallstones) which also included 48 cases with primary sclerosing cholangitis were reviewed systematically by the authors for any polyp ≥2 mm. Also reviewed specifically for this purpose by histopathology was a gallbladder cancer cohort composed of 606 cholecystectomies with gallbladder carcinomas. All diagnostic slides were reviewed and categorized based on the criteria described above.

Of the participating institutions, those from Chile are referral centers for both gallstone disease as well as associated gallbladder cancers, and in fact, one of the participating sites in Chile (Temuco) currently serves as a referral center at a region that has one of the highest incidences of gallbladder cancer in the world [26]; most of the 606 gallbladder cancer cases evaluated for polyp in this study were from this site. Of note, all case-contributing institutions serve both as primary care as well as referral centers and are located in major cities of the respective countries and thus present a mixture of both routine and complicated patients.

The findings were correlated with the clinicopathologic parameters. Clinical information (age, gender) was obtained through pathology databases.

**Fig 3:**
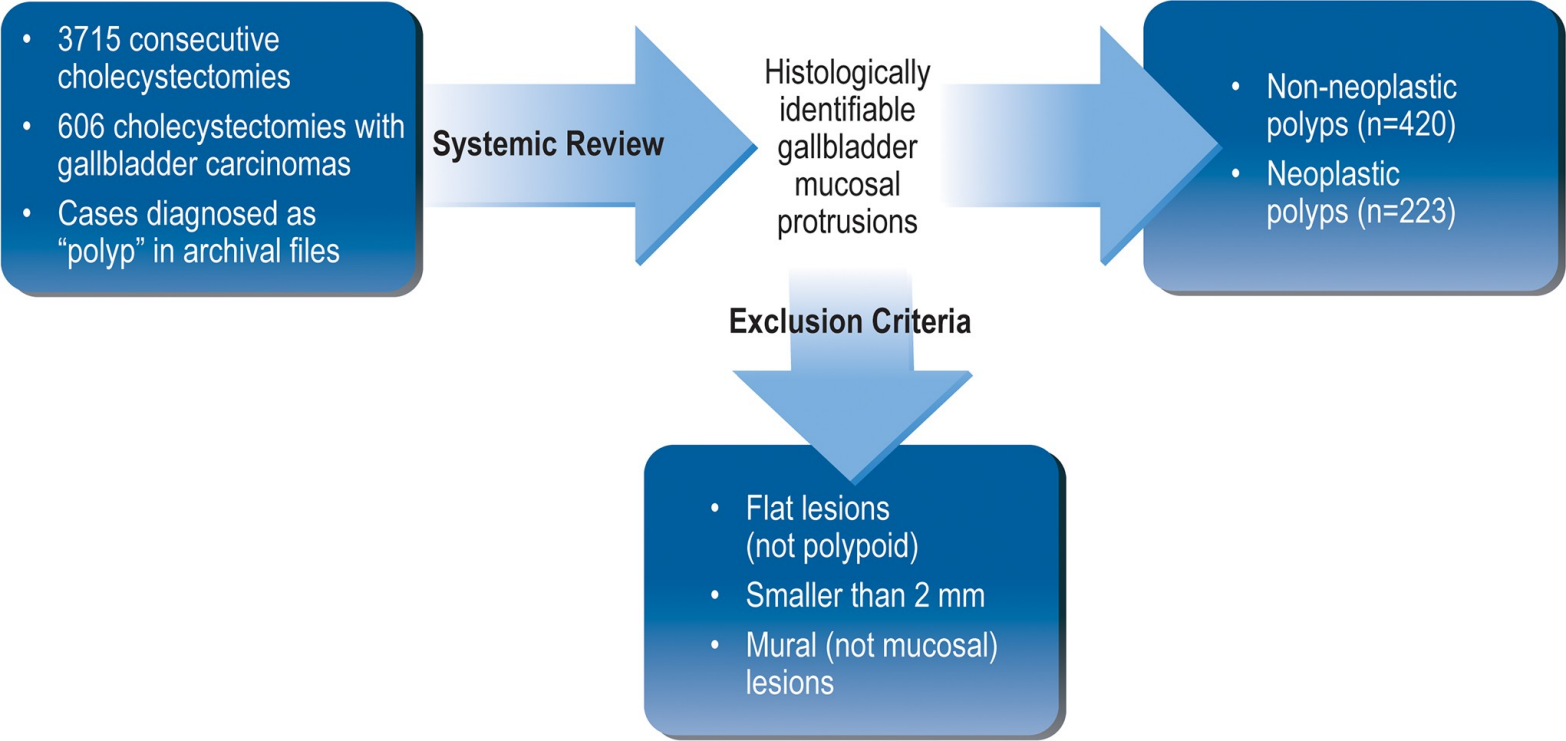
The flowchart summarized inclusion and exclusion criteria.

In Fig 3, inclusion and exclusion criteria were summarized in a flowchart.

### Statistical analysis

Descriptive statistics were given as mean, median, standard deviation, and range. Normality of continuous data was evaluated by using Shapiro Wilks Test. Mann-Whitney U test was used to compare size differences between neoplastic and non-neoplastic polyps. For comparison of more than two independent non-normally distributed groups Kruskal Wallis test was used. As Post-Hoc analysis Bonferroni corrected Mann-Whitney U test was used. A p-level of 0.05 was accepted as significant.

Diagnostic test calculations (sensitivity, specificity, positive predictive value and negative predictive value) for polyp size were calculated while the presence of neoplastic polyp taken as gold standard. The optimal cut-off for polyp size for this cohort was determined using ROC analysis and Youden index. Additionally, a 10 mm cut-off was used to form contingency table to assess diagnostic test calculations.

ROC analysis was performed by R-project [R Core Team (2019)]. R: A Language and environment for statistical computing [Computer software, version 3.6.0]retrieved from https://cran.r-project.org/), and pROC package (BMC Bioinformatics, 12, p. 77. doi: 10.1186/1471-2105-12-77). Mann-Whitney U test was performed with the jamovi project (2019); jamovi (Version 1.1) [Computer Software] retrieved from https://www.jamovi.org. MedCalc Diagnostic test evaluation calculator (https://www.medcalc.org/calc/diagnostic_test.php) was used for sensitivity and specificity calculation.

## Results

### I. Clinicopathologic associations of different polyp types

#### All polyps (n = 643)

Mean age of patients was 55 years (range: 20–94). Mean polyp size was 9 ± 11 mm. Median polyp size was 4 mm (IQR: 7) (range: 2–77). 420 and 223 cases were classified as non-neoplastic and neoplastic polyps, respectively.

*I*. *Non-neoplastic polyps (n = 420; 65*.*4%)*. Non-neoplastic polyps consisted of fibromyoglandular polyps (n = 196), cholesterol polyps (n = 166), polypoid pyloric gland metaplasia (n = 41) and inflammatory polyps (n = 17). Mean age of patients was 52 years (range: 21–93). Mean polyp size was 4.1 ± 2.3 mm. Median polyp size was 3.5 mm (IQR: 2).

There was a statistically significant difference in size between different non-neoplastic polyp groups (p<0.001): In post-Hoc analysis, a statistically significant difference was found between cholesterol polyps vs. fibromyoglandular polyps (p = 0.001) and fibromyoglandular polyps vs. polypoid pyloric gland metaplasia (p = 0.002). Detailed clinicopathologic features and size distribution of each diagnostic subgroup were shown in Table 1.

**Table 1:** Clinicopathologic features and size distribution of gallbladder polyps.

	Polyp Types (defined as ≥ 2mm)	n	Mean age years (range)	Sex (F/M)	Mean size ± SD (mm)	Median size (IQR) (mm)	≥1 cm		≥2 cm	
**Non-neoplastic** **polyps (n = 420)**	**Cholesterol polyp**	166	46 (21–76)	2.4	3.9 ± 2.4	3 (2)	6 (4%)	17 (4% of all NNPs)	0	1 (0.2% of all NNPs)
	**Fibromyoglandular polyp**	196	55 (23–93)	4.4	4.2 ± 2	4 (2)	9 (5%)		0	
	**Polypoid pyloric gland metaplasia**	41	55 (24–77)	3	3.3 ± 1.4	3 (2)	0		0	
	**Inflammatory polyp**	17	59 (38–85)	1.8	6 ± 4.5	6 (4)	2 (12%)		1 (6%)	
**Neoplastic polyps (n = 223)**	**Intracholecystic neoplasm**	120	61 (20–94)	2	26.3 **±** 14.2	22 (20)	120 (by definition)	155 (69.5% of all NPs)	73 (61%)	93 (41% of all NPs)
	**Incipient (<1 cm) intracholecystic neoplasm**	44	59 (36–83)	6.4	4.1 **±** 1.8	3 (2)	0 (by definition)		0 (by definition)	
	**Polypoid invasion**	26	71 (48–88)	7.3	28 **±** 13	27 (17.25)	25 (96%)		18 (69%)	
	**NNP with dyplasia**	33	57 (37–83)	2	8 **±** 9	5 (7)	10 (30%)		2 (6%)	

NP: Neoplastic polyp.

NNP: Non-neoplastic polyp.

*II*. *Neoplastic polyps (n = 223; 34*.*6%)*. The largest group in this category was intracholecystic neoplasms (n = 164, with 120 of these ≥1 cm, and 44 <1 cm, i.e., “incipient”). There were 26 polypoid invasive carcinomas. The remainder were non-neoplastic polyps that harbored dysplastic change (n = 33; eight were high-grade dysplasia/in-situ carcinoma and the remainder were lesser grade lesions). Mean age of patients was 61 years (range: 20–94). Mean polyp size was 19 ± 15.4 mm. Median polyp size was 15 mm (IQR: 23).

In addition to polypoid carcinomas that were invasive by definition, an invasive carcinoma component was observed in 49% (n = 80/164) of intracholecystic neoplasms and 9% (n = 3/33) of non-neoplastic polyps that harbored dysplastic changes.

There was a statistically significant difference in size between different neoplastic polyp groups (p<0.001): In post-Hoc analysis, a statistically significant difference was found between intracholecystic neoplasms vs. non-neoplastic polyps with dysplasia (p = 0.001) and non-neoplastic polyps with dysplasia vs. polypoid invasion (p = 0.001). Detailed clinicopathologic features and size distribution of each diagnostic subgroup were shown in Table 1.

### II. Relationship of size and neoplastic change

Overall, 172 of 643 cases (27% of all polyps) were ≥1 cm. Among these, 155 (90%) were neoplastic (neoplastic polyps and non-neoplastic polyps with at least focal dysplastic/neoplastic changes) and 17 (10%) were non-neoplastic. Among 471 polyps measuring <1 cm, 403 (86%) and 68 (14%) were non-neoplastic and neoplastic, respectively. There was only one non-neoplastic polyp, an inflammatory polyp consisting of granulation tissue, measuring ≥2 cm. Positive predictive value of ≥1 cm cut-off for the presence of neoplasm was 94.3% and negative predictive value was 84.7%.

The mean size of all polyps examined histopathologically (and measured ≥2 mm per the study criteria) was 9 mm. Non-neoplastic polyps were significantly smaller (mean size: 4.1 mm) than neoplastic ones (mean size: 21 mm) (p<0.001). From the neoplasia perspective, 70% of the neoplastic polyps were ≥1 cm and 30% were smaller. Among those that were <1 cm, most (65%, n = 44/68) were “incipient” intracholecystic neoplasms, a third (34%, 23/68) was non-neoplastic polyps with dysplasia and only one was polypoid invasive carcinoma. See Table 2 for detailed information.

**Table 2:** Distribution of cases based on 10-mm size cut-off.

	Size <10 mm (n)	Size ≥10 mm (n)	Total (n)
**Non-neoplastic polyps (n)**	403 (96% of all NNPs) (63% of all polyps)	17(4% of all NNPs)(3% of all polyps)	420
**Neoplastic polyps (n)**	68 (30% of all NPs) (10% of all polyps)	155(70% of all NPs)(24% of all polyps)	223
**Total (n)**	471 (85% NNP, 15% NP)	172 (90% NP, 10% NNP)	643

Among neoplastic polyps, the presence of an invasive component was significantly correlated with bigger size [mean size 25.1 (±15.4) mm in those with an invasive component vs. 13.9 (±13.4) mm in those without an invasive component, p<0.001).

94 of 643 cases (15% of all polyps) were ≥2 cm. Among 94 cases that were ≥2 cm, 93 (99%) were neoplastic, comprising of INs (n = 73), polypoid invasive carcinomas (n = 18) and non-neoplastic polyps with dysplasia (n = 2). In this group, the only non-neoplastic polyp (1%) was a 20 mm inflammatory polyp (granulation tissue polyp).

### Size cut-off

**Fig 4:**
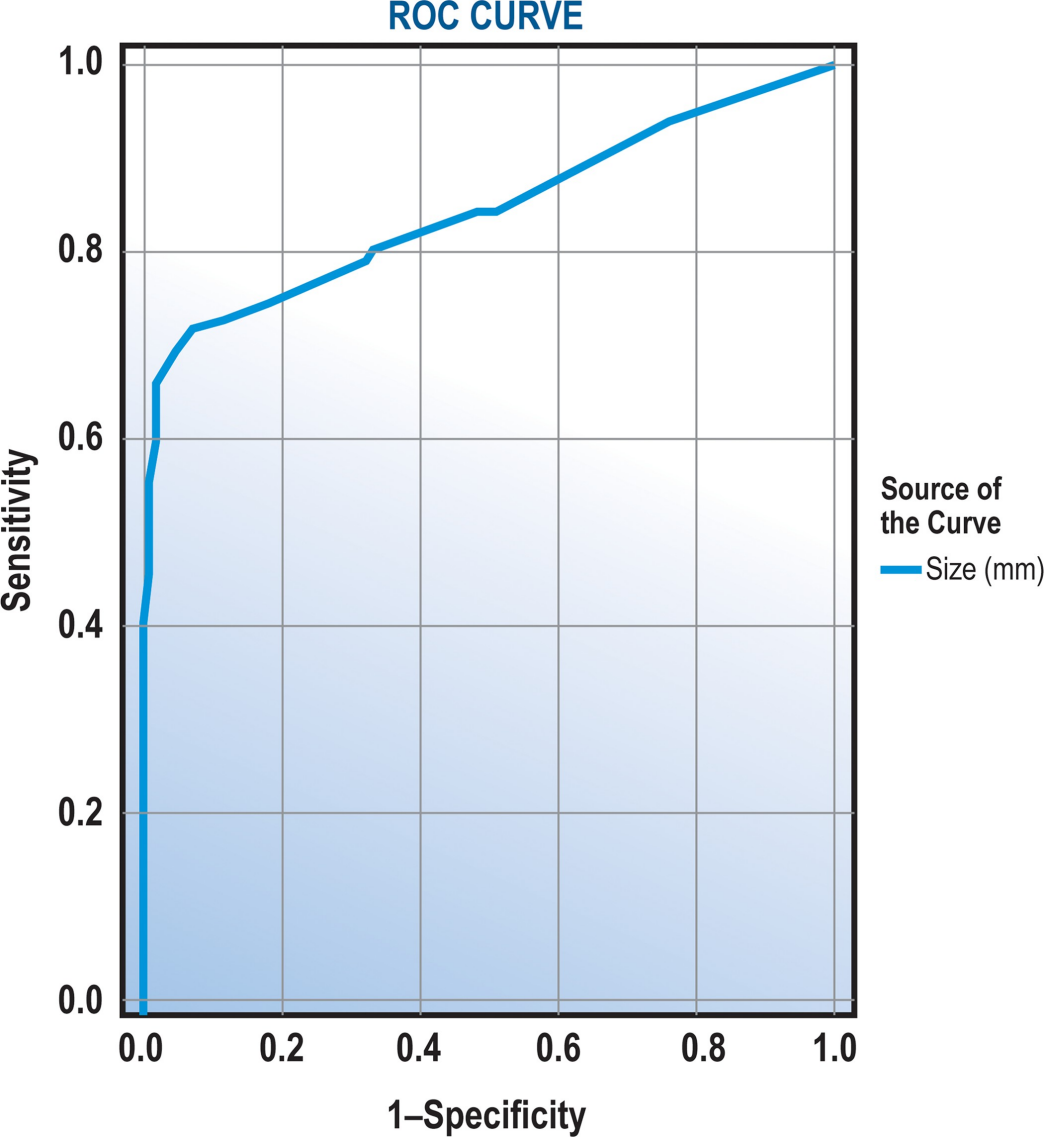
The ROC analysis has revealed a cut-off of 9 mm, with AUC = 0.854 [0.818–0.889, 95% CI]. At this threshold, the sensitivity was 69.5% and specificity was 95.9%.

Sensitivity of >1 cm for the neoplasia is 66.8% [60.2%-73%, 95%CI], and specificity is 97.8% [96%-99%, 95%CI]. The positive predictive value of >1 cm in predicting neoplasia is 94.3% [89.5%-97.4%, 95%CI]. In accordance with the literature, in our cohort, the ROC analysis has revealed a very close cut-off, namely 9 mm, with AUC = 0.854 [0.818–0.889, 95% CI] (See Fig 4). Similarly, at this threshold, the sensitivity was 69.5% and specificity was 95.9%. Table 3 shows sensitivity and specificity of different cut-off points.

**Table 3:** Sensitivity, specificity, positive and negative predictive values of different cut-off points.

Criterion	Sensitivity (%) (95% Cl)	Specificity (%) (95% Cl)	Positive Predictive Value (95% Cl)	Negative Predictive Value (95% Cl)
>5 mm	74.4 (68.2 - 80)	83.9 (80.1 – 87.4)	71.2 (65 – 77)	86 (82.3 – 89.2)
> 9 mm	69.5 (63–75.5)	95.9 (93.6–97.6)	90.1 (84.6–94.1)	85.5 (82–88.6)
>10 mm	66.8 (60.2 - 73)	97.8 (96 - 99)	94.3 (89.5 – 97.4)	84.7 (81.2 – 87.8)
>15 mm	49.7 (43 – 56.5)	99.2 (97.9 – 99.9)	97.4 (92.5 – 99.5)	78.7 (75 – 82.2)
>20 mm	37.6 (31.3 – 44.4)	100 (99.1 - 100)	100 (95.7 – 100)	75 (71.2 – 78.6)
>25 mm	29.6 (23.7 – 36.1)	100 (99.1 - 100)	100 (94.6 – 100)	72.7 (68.9 – 76.3)

## Discussion

Pre-operative determination of the nature of gallbladder lesions is fraught with challenges. For gallbladder polyps, size measurement has been used as the simplest way to estimate the potential nature of the lesion and to determine the course of action, since ultrasonography is the most common and accessible method for their radiologic evaluation [2,27]. One centimeter is currently the most commonly used rule-of-thumb criteria for a polyp to be removed, although data substantiating this cut-off has been debated [15]. Part of the challenge is that most of the studies to date investigated this issue based on the selective cholecystectomies performed [10–13,16,27–34], which inevitably included biased cohorts and cases that may have been misinterpreted as polyps radiologically [15]. For example, the conditions that are by default mural nodular lesions rather than being true mucosal polyps such as adenomyomas and xanthogranulomatous cholecystitis, both of which cause thickening of gallbladder wall and understandably lead to the erroneous diagnosis of a polypoid lesion were included in the studies as “polyps” [16]. In contrast, in this study, this question was addressed from the perspective of polypoid lesions in a large cohort of cholecystectomy performed for a variety of causes, including cancers, in order to determine the relative frequency of true mucosal polyps, their classification and clinicopathologic associations from the pathology perspective.

This study elucidated that 35% (223/643) of the true polypoid lesions identified histopathologically that are ≥2 millimeters in cholecystectomies performed for variety of causes prove to be neoplastic. The vast majority of these neoplastic polyps are entities that warrant serious and prompt attention. For example, a not too trivial percentage of these are in fact invasive adenocarcinomas that have a prominent polypoid growth (11% of the neoplastic polyps and 4% of all polyps). Of the remainder, most neoplastic polyps are intracholecystic neoplasms (pyloric gland adenomas and intracholecystic papillary neoplasms, which are collectively called intracholecystic papillary tubular neoplasms). These are known to have high propensity to be associated with invasive carcinoma in the same gallbladder or progress into invasion in follow up [9]. Therefore, accurate diagnosis and management of these cases are crucial.

In terms of the size correlation, more than 2/3^rd^ of neoplastic polyps are larger than 1 cm. Along those lines, of the polyps that are >1 cm, 90% are neoplastic. This is because while non-neoplastic polyps are a lot more common (>70% of the polyps from pathology perspective), they generally remain fairly small with a median size of 3.5 mm (IQR: 2). Additionally, only about 1 in 6 non-neoplastic polyps (14%) achieve a size of 1 cm or above. When it comes to 2 cm, virtually all polyps of this minimum size are neoplastic; there was only one exception in our cohort, an inflammatory/granulation tissue polyp resembling pyogenic granuloma that was 2 cm. Therefore, a convincing mucosal polyp that is >2 cm ought to be regarded neoplastic for all practical purposes.

Thus, this study supports, from pathology perspective, an important aspect of the criteria put forth by radiology-pathology correlation studies -that is now also incorporated into the guidelines- that gallbladder polyps ≥1 cm indeed warrant cholecystectomy [2]. The positive predictive value of the 1 cm cut-off for the presence of neoplasm was 94.3% and negative predictive value, 85%. In fact, statistical methods highlighted the size of 9 millimeters as the “sweet spot” balancing the predictive values for the identification and exclusion of a neoplastic polyp. However, this study also illustrates that polyps smaller than 1 cm should not generate the assurance that seems to be the impression given in the literature: Nearly a third of the neoplastic polyps were actually <1 cm. Granted, when the entire population is considered, the number of neoplastic polyps in gallbladder that measure <1 cm are fairly small, nevertheless, at the same time, it is those rare cases that could benefit tremendously from the removal of the polyp. As radiologic methods become more accurate and more widely used in the general population, this will become even more common and important. Therefore, small polyps also warrant close follow-up and perhaps more advanced radiologic analysis. Especially if the patient is older, such a case may have to be investigated with further attention. More studies are required to determine the radiologic and clinical correlates of true gallbladder polyps highlighted in this study. Now that the polypoid lesions are better characterized at the histopathologic level, their reflection at the radiologic level will be more appreciable with proper pathology-radiology correlation studies. For example, cholesterol polyps have very distinctive morphology at the microscopic level. Considering they also are rich in fat, additional radiologic evaluation techniques focusing on the fat content have the potential to be very useful in determining the nature of the polyp and establishing the course of action.

In summary, from pathology perspective, about a third of the true mucosal polyps in the gallbladder are neoplastic in nature. In terms of associations with size, the vast majority of the polyps (90%) that are larger than 1 cm are indeed neoplastic and therefore, the current approach of using 1 cm as the cut-off is highly applicable. However, at the same time, a not too trivial percentage of neoplastic lesions are smaller than 1 cm, and for this reason, smaller polyps also need to be observed closely to rule out a neoplastic process, especially if it is in an older patient. More studies are needed to establish criteria for the pre-operative diagnosis of the true mucosal polyps and to determine the progression risk of polypoid lesions less than 1 cm and selecting them for better management.
