# Correction: Gallbladder polyps: Correlation of size and clinicopathologic characteristics based on updated definitions

**DOI:** 10.1371/journal.pone.0306997

**Published:** 2024-07-05

**Authors:** Orhun C. Taskin, Olca Basturk, Michelle D. Reid, Nevra Dursun, Pelin Bagci, Burcu Saka, Serdar Balci, Bahar Memis, Enrique Bellolio, Juan Carlos Araya, Juan Carlos Roa, Oscar Tapia, Hector Losada, Juan Sarmiento, Kee-Taek Jang, Jin-Young Jang, Burcin Pehlivanoglu, Mert Erkan, Volkan Adsay

After publication of this article [1], concerns were raised about typographical errors and the interpretation of the analysis. A statistical reviewer and members of the *PLOS ONE* Editorial Board reviewed the article and the concerns and indicated that there were errors and omissions in the reporting of the Methods and Discussion, and the description of the statistical analysis.

The article describes the statistical analyses as a correlation analysis. However, the approach used is only able to measure statistical association. Therefore, the terms “correlation(s)”, and “correlated” are corrected to “association(s)” and “associated” throughout the article, including the Title and Abstract. This does not affect the article conclusions.

There is a typographical error in the Abstract. In the first sentence of the Methods section, 4231 is corrected to 4,321. This has no effect on the statistical analysis or article conclusions.

There are typographical errors in the article with respect to the use of “greater than” symbols, since the size distributions reported in Tables 1 and 2 are based on polyp sizes greater than or equal to the cut-off criterion, whereas diagnostic test calculations reported in Table 3 are based on polyp size greater than the cut-off criterion. The following corrections are made:

- “≥1 cm” in the last sentence of the Results section of the abstract, and the last sentence of the first paragraph of section II of the Results section of the main manuscript is corrected to “>10 mm”.
- “larger than 1 cm” in the first sentence of the third paragraph, and the second sentence of the last paragraph of the Discussion is corrected to “1 cm or larger”.
- “>1 cm” in the second sentence of the third paragraph of the Discussion is corrected to “*≥*1 cm”.

The authors also clarify that these errors have no effect on the statistical analysis or article conclusions, and the 1 cm cut-off in the literature is variably reported as both >1 cm and ≥1 cm.

The Methods could be misinterpreted to imply that the definition of a polyp changed over time. Therefore, the following is added to the end of the second paragraph of the Definition of polyp, inclusion criteria, selection of size cut-off section: “This same definition was applied to all cases.”

There are omissions from the Case population/databases section of the Methods. Therefore, the first two paragraphs of this section are replaced by the following:

“Pathological specimens were sourced from the institutional surgical pathology files of two institutions in Chile and the USA. In addition to institutional material, consultation material received by authors (JCR and VA) for a second opinion from institutions around the world was included. Files and reports from 1994 to 2013 were available.

The center in Chile was Universidad de la Frontera, in Temuco, Chile. This institution resides in a region where gallbladder cancer is one of the highest in the world [26]. It serves as the referral center for various smaller hospitals in the broader region that refer all their cholecystectomy specimens to this pathology department for examination per government insurance protocols. In other words, these gallbladders had been removed in various hospitals in the central region of Chile and sent to Universidad de La Frontera in Temuco.

The cases from the United States were from the Emory University Hospitals (Emory Healthcare), Atlanta, GA. This is one of the largest multi-hospital complexes in the United States, comprising Emory University Hospital, Emory University Hospital-Midtown (previously called Crawford-Long Hospital), and Grady Memorial Hospital. It is the only major University-based academic medical center in metropolitan Atlanta, serving a metropolis of about 6 million people. It also serves as both a primary and tertiary care center. In the United States, half a million to a million gallbladders are removed annually, and major academic centers like Emory Healthcare receive a higher share of these. Emory also has very active hepatobiliary surgery and liver transplant services. The institution has a strong policy of second-opinion review of patients’ prior pathology material by the Emory pathology department before definitive therapy is instituted. All of these factors lead to a substantial number of gallbladder specimens with variable pathology being reviewed in this medical complex.

A total of 4,321 gallbladder specimens, of which 606 consisted of cholecystectomies with gallbladder carcinomas, were pathologically reviewed by the authors to identify polypoid lesions, regardless of whether polyps had previously been diagnosed or not. All pathological material and polyps were examined by the authors according to criteria established during in-person meetings before the study, as described above. For the polyps identified, all cases from Chile were reviewed by EB, JCA, JCR, and all cases from the US were reviewed by VA and ND. Additionally, the authors met numerous times to discuss uncertain cases (in Temuco, Chile, and Santiago, Chile, with the participation of VA, JCR, JCA, OB, MDR, and EB, and in Atlanta, GA, with the participation of VA, MDR, and JCR). All the authors have expertise in gallbladder pathology.

In addition, a computer search of pathology archives for cases that had been recorded as “polyp” in routine diagnostic workup by institutional pathologists was conducted. This source facilitated the identification of cases that had been recorded pathologically as having a “polyp” among tens of thousands of cholecystectomies, which are not included among the 4,321 gallbladder specimens mentioned above. In other words, this component of the study group relied on the original diagnoses in the archives of the institutions for initial identification. Any case that had been recorded as a “polyp” was retrieved and examined by the authors to verify the existence of a polyp that fit the inclusion criteria. Only cases with qualified polyps were included in the study for further evaluation. The cases that did not qualify were not specifically recorded since this issue (mis-coding, mis-classifications, or other causes) was not a specific goal of this study.”

There are omissions in Fig 3 describing how cases were selected for inclusion in the study. An updated Fig 3 is provided here.

The article does not include supporting statistics for claims in the Discussion about the particular need to monitor smaller polyps in older populations. Therefore, the following is added to the Results:

- The following sentence is added after the first sentence of the All polyps section of the Results: “Mean age of the Non-neoplastic Polyps group versus Neoplastic Polyps was 52 vs 61, p< 0.001”.
- The following sentence is added after fifth sentence of the first paragraph of Neoplastic polyps section of the Results: “Mean age of the Non-neoplastic Polyps group versus Polypoid Invasive Carcinomas (full blown cancer cases) was 52 vs 71, p< 0.001”.

There are omissions in the discussion of the limitations of the study. Therefore, the following paragraph is added between the fourth and fifth paragraphs of the Discussion:

“Although a strength of this study is the large number of cases as well as their careful pathologic review (also involving international consensus meetings), there are limitations to this study. Ultimately this is a design-wise simple documentative study, investigating the characteristics of gallbladder polyps from pathology perspective and how they relate to size. Cross-validation of the results would be required to further explore the generalizability of the study findings. In addition, there is often some degree of shrinkage, which may influence the size measurement of a lesion and create a discrepancy between radiology and pathology. However, this discrepancy may not be at a magnitude to impact the daily practice dramatically.”

Concerns were raised that although the ROC curve analysis yielded an optimal cut-off value of 9 mm for polyp diameters, the Discussion focuses on polpys ≥1 cm. The authors clarify that their ROC analysis result, being very close to the arbitrary criterion widely used to determine indication for cholecystectomy of polyps ≥1 cm, provides support for the validity of this practice. However, they state it would have been inappropriate to use this result to suggest changes to the established cut-off employed in daily practice.

Concerns were raised that [1] appears to imply that conclusions can be made about the incidence of polyp formation, but the study design cannot support such conclusions. The authors clarify that the article does not attempt to analyze the epidemiological incidence of polyp formation. Instead, all the percentages mentioned in the article are based on the findings of the specific cohort being studied. The following corrections are made:

- The last sentence of the Abstract is corrected to: “However, 30% of the neoplastic polyps included in this study are <1 cm and therefore small polyps should also be closely watched, especially in older patients.”
- The first sentence of the second paragraph of the Discussion is corrected to “In this study, 35% (223/643) of the true polypoid lesions identified histopathologically that are ≥2 millimeters in cholecystectomies performed for variety of causes are found to be neoplastic.”
- The first sentence of the third paragraph of the Discussion is corrected to “In terms of the size correlation, more than 2/3^rd^ of neoplastic polyps in this study are 1 cm or larger.”
- The first sentence of the fifth paragraph of the Discussion is corrected to: “In summary, from pathology perspective, about a third of the true mucosal polyps in the gallbladder included in this study are neoplastic in nature.”

In addition, the primary data underlying results in this article were not included with the published article although the Data Availability statement for this article stated, “All relevant data are within the paper.” in error. With this Correction, the authors provide the original raw data via OSF Repository, and update the Data Availability statement to: “All relevant data are available in the OSF Repository https://osf.io/dr3cf/.”

During the course of this investigation, it was noted that the original handling Academic Editor had recently co-published a number of articles with some of the authors at the time of peer review. A second member of *PLOS ONE*’s Editorial Board has re-evaluated the manuscript and has confirmed that, with the above corrections, the article is scientifically sound and meets *PLOS ONE*’s Publication Criteria.

**Fig 3:**
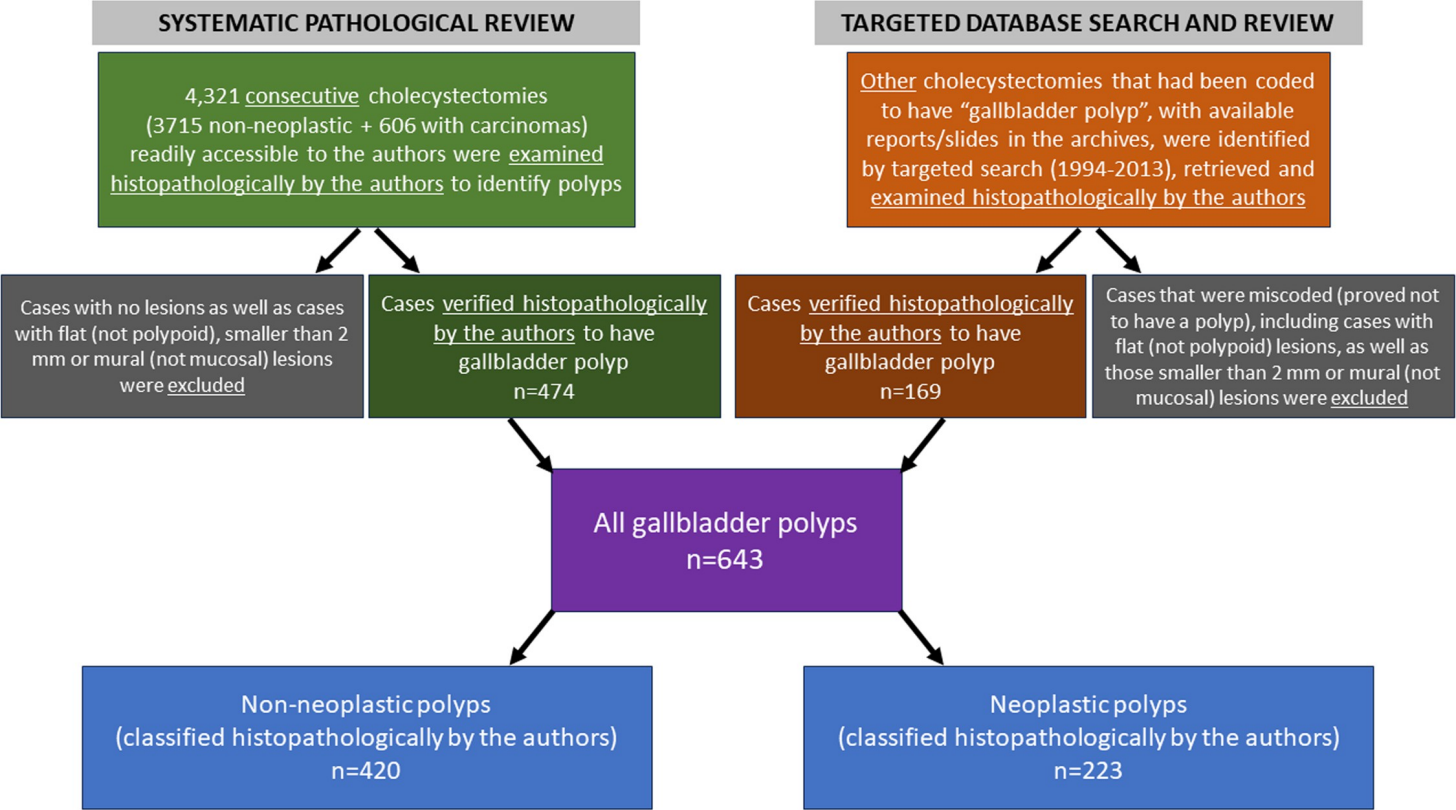
The flowchart summarizing inclusion and exclusion criteria.
